# Enteric bacterial infection in *Drosophila* induces whole-body alterations in metabolic gene expression independently of the immune deficiency signaling pathway

**DOI:** 10.1093/g3journal/jkac163

**Published:** 2022-07-04

**Authors:** Rujuta Deshpande, Byoungchun Lee, Savraj S Grewal

**Affiliations:** Clark H Smith Brain Tumour Centre, Arnie Charbonneau Cancer Institute, Alberta Children’s Hospital Research Institute, University of Calgary, Alberta T2N 4N1, Canada; Department of Biochemistry and Molecular Biology Calgary, University of Calgary, Alberta T2N 4N1, Canada; Clark H Smith Brain Tumour Centre, Arnie Charbonneau Cancer Institute, Alberta Children’s Hospital Research Institute, University of Calgary, Alberta T2N 4N1, Canada; Department of Biochemistry and Molecular Biology Calgary, University of Calgary, Alberta T2N 4N1, Canada; Clark H Smith Brain Tumour Centre, Arnie Charbonneau Cancer Institute, Alberta Children’s Hospital Research Institute, University of Calgary, Alberta T2N 4N1, Canada; Department of Biochemistry and Molecular Biology Calgary, University of Calgary, Alberta T2N 4N1, Canada

**Keywords:** *Drosophila*, transcriptome, enteric infection, metabolism, Imd pathway, transcription factors, neuropeptides

## Abstract

When infected by intestinal pathogenic bacteria, animals initiate both local and systemic defence responses. These responses are required to reduce pathogen burden and also to alter host physiology and behavior to promote infection tolerance, and they are often mediated through alterations in host gene expression. Here, we have used transcriptome profiling to examine gene expression changes induced by enteric infection with the Gram-negative bacteria *Pseudomonas entomophila* in adult female *Drosophila*. We find that infection induces a strong upregulation of metabolic gene expression, including gut and fat body-enriched genes involved in lipid transport, lipolysis, and beta-oxidation, as well as glucose and amino acid metabolism genes. Furthermore, we find that the classic innate immune deficiency (Imd)/Relish/NF-KappaB pathway is not required for, and in some cases limits, these infection-mediated increases in metabolic gene expression. We also see that enteric infection with *Pseudomonas entomophila* downregulates the expression of many transcription factors and cell–cell signaling molecules, particularly those previously shown to be involved in gut-to-brain and neuronal signaling. Moreover, as with the metabolic genes, these changes occurred largely independent of the Imd pathway. Together, our study identifies many metabolic, signaling, and transcription factor gene expression changes that may contribute to organismal physiological and behavioral responses to enteric pathogen infection.

## Introduction

When infected with pathogenic bacteria, animals need to mount appropriate defence responses to promote their survival. Perhaps the best-studied mechanisms are the innate immune responses that are involved in sensing invading pathogens and initiating antibacterial responses (Buchon *et al.* 2014). These mechanisms are termed resistance responses and are initiated to reduce pathogen burden. Recent work has also shown how bacterial infections can induce systemic changes in physiology and metabolism (Medzhitov *et al.* 2012; Martins *et al.* 2019; Troha and Ayres 2020). These metabolic changes can be required to fuel the energetically costly innate immune resistance responses to bacterial infection (Ryan and O’Neill 2020). In some cases, however, these changes can promote infection survival without reducing pathogen levels. These are termed tolerance responses and often involve changes in host metabolism, physiology, and behavior (Ayres and Schneider 2012; Medzhitov *et al.* 2012) that function to limit the damaging effects of the pathogen and maintain host health. While these tolerance responses are not as well studied as the classic innate immune resistance responses, it is becoming clear that they are as important in determining individual susceptibility to infection (Ayres 2020).

*Drosophila* has been a versatile and informative model system in the investigation of organismal defence responses to bacterial infection (Younes *et al.* 2020). Oral infection of adult flies with Gram-negative pathogenic bacteria induces both local and systemic resistance responses. Central to these responses is induction of the Imd/NF-Kappa B signaling pathway (Kleino and Silverman 2014). This pathway senses bacteria through cell surface peptidoglycan recognition protein (PGRP) receptors and signals through an intracellular cell signaling pathway involving the death domain containing protein, Imd, leading to transcriptional activation of the Relish/NF Kappa B transcription factor (Capo *et al.* 2016). One main target of Relish are antimicrobial peptides that mediate resistance responses to reduce pathogen load (Buchon *et al.* 2014). Enteric Gram-negative bacterial infection in *Drosophila* can also induce both local and systemic changes in host physiology and metabolism to promote both resistance and tolerance responses (Lee and Lee 2018; Galenza and Foley 2019; Bland 2022). For example, gut infection induces altered lipid storage and metabolism both in the intestine and in remote tissues such as fat body and oenocytes (Hang *et al.* 2014; Charroux *et al.* 2018; Kamareddine, Robins, *et al.* 2018; Kamareddine, Wong, *et al.* 2018; Lee *et al.* 2018; Harsh *et al.* 2019; Zhao and Karpac 2021; Charroux and Royet 2022; Deshpande *et al.* 2022). Enteric infection can also induce alterations in systemic carbohydrate, mitochondrial, and amino acid metabolism (Zhao and Karpac 2021). These nonautonomous effects of enteric infection on whole-body metabolism involve changes in both gut-to-fat and muscle-to-fat metabolic signaling and are required to promote host fitness. Enteric bacterial infection can also cause changes in fly behavior that may help to promote infection tolerance, such as food avoidance and reduced fecundity (Soldano *et al.* 2016; Masuzzo *et al.* 2019, 2020; Charroux *et al.* 2020). In some cases, these effects have been shown to occur as a result of altered gut-to-brain signaling (Cai *et al.* 2021). Together, these studies show that gut pathogens can trigger tissue-to-tissue signaling in flies to coordinate changes in metabolism, physiology, and behavior and promote both resistance and tolerance responses to infection. Understanding the mechanisms underlying these changes will provide further insights into how organismal defence responses.

One way that organismal physiology can be altered is through changes in gene expression. To provide further insight into systemic responses to enteric infection, we have used transcriptome profiling to identify whole-body gene expression changes following enteric infection with *Pseudomonas entomophila* (*P.e*). Our data reveal upregulation of many metabolic genes involved in lipid, carbohydrate, and amino acid metabolism, and we see that induction of these genes occurs independently of Imd signaling and, in some cases, is antagonized by the Imd signaling pathway. In addition, we saw a downregulation of genes encoding transcription factors, signaling peptides, and signaling receptors, particularly those involved in gut-to-brain and neuronal signaling. Together, these analyses reveal broad changes in metabolic, signaling, and transcription factor gene expression that may mediate organismal responses to infection.

## Materials and methods

### *Drosophila* stocks and culturing

The following strains were used: *w^1118^, imd^[EY08573]^* (Bloomington stock center no. 17474). Flies were grown on medium containing 150 g agar, 1,600 g cornmeal, 770 g Torula yeast, 675 g sucrose, 2,340 g D-glucose, 240 ml acid mixture (propionic acid/phosphoric acid) per 34 l water. All stocks were maintained at either 18°C or RT. For infection experiments, flies were raised from embryos to adults at 25°C. Following eclosion, females were allowed to mate for 2 days before being separated from males and aged for another 5–6 days, at which time infection experiments were performed. All experiments were conducted in mated adult females. For the genetic experiments, *w^1118^* flies were used as the controls because our *imd^[EY08573]^* stock was maintained in a *w^1118^* background.

### Enteric infections

Enteric infections were performed using previously described methods (Buchon *et al.* 2010; Zhao and Karpac 2021). Briefly, *P.e* from overnight cultures were pelleted and resuspended in 5% sucrose solution (in PBS) such that the final concentration of bacteria was OD_600_ = 200. Bacterial pellets were then dissolved in filter sterilized 5% sucrose/PBS. Chromatography paper (Fisher, Pittsburgh, PA) discs were dipped in the bacterial solution and were carefully placed on standard fly food vials such that they covered the entire food surface. Adult females were first subjected to a 2-h starvation period in empty vials at 29°C. Then 10–12 flies were transferred to each infection vial and then placed in a 29°C incubator for the duration of the 24-h infection period. Mock-infected control flies were placed in similarly prepared vials that contained paper discs soaked in 5% sucrose/PBS alone.

### Total RNA isolation

Adult flies were snap frozen on dry ice in groups of 5. Total RNA was then isolated using Trizol according to the manufacturer’s instructions (Invitrogen; 15596-018). Extracted RNA was then DNase treated (Ambion; 2238G) to be used for subsequent qPCR or mRNA sequencing.

### mRNA sequencing

Six independent biological replicates (5 flies per group) of mock infected and *P.e.* infected groups were prepared and analyzed. RNA-sequencing was conducted by the University of Calgary Centre for Health Genomics and Informatics. The RNA Integrity Number (RIN) was determined for each RNA sample (6 replicates per each condition were used). Samples with a RIN score higher than 8 were considered good quality, and Poly-A mRNA-seq libraries from such samples were prepared using the Ultra II Directional RNA Library kit (New England BioLabs) according to the manufacturer’s instructions. Libraries were then quantified using the Kapa qPCR Library Quantitation kit (Roche) according to the manufacturer’s directions. Finally, RNA libraries were sequenced for 100 cycles using the NextSeq 500 Sequencing System (Illumina).

### RNA-seq analyses

Quality control of sequenced DNA was carried out using FastQC. Sequenced reads were mapped to the *Drosophila* genome Release 6 (GCF_000001215.4_Release_6_plus_ISO1_MT_rna) and transcripts were quantified using kallisto (Bray *et al.* 2016). Measurements of differential expression were made using sleuth (Pimentel *et al.* 2017).

### Gene ontology, KEGG pathway, and tissue expression analyses

Analyses of Gene Ontology and KEGG pathway enrichment of up- and downregulated genes [>1.5-fold, *q*-val (FDR corrected *P*-val) <0.05] were performed using G-profiler (Raudvere *et al.* 2019) and Revigo (Supek *et al.* 2011). Tissue enrichment of differentially expression in Figs. 5 and 6 was examined using FlyAtlas2 (Leader *et al.* 2018). We defined genes having enrichment in head and brain (combined expression levels in adult head, eye, brain/CNS, thoracicoabdominal ganglion) and/or intestinal system (combined expression levels in crop, midgut, and hindgut) if expression in these tissues was >3-fold higher than any other tissue(s).

### Quantitative RT–PCR measurements

Total RNA was extracted from either whole flies or from isolated intestines or abdominal samples (abdominal carcass containing attached fat body, but with ovaries, guts, and Malpighian tubules removed). The RNA was then DNase treated as describe above and reverse transcribed using Superscript II (Invitrogen; 100004925). The generated cDNA was used as a template to perform qRT–PCRs (ABI 7500 real time PCR system using SyBr Green PCR mix) using gene-specific primers. PCR data were normalized to *5S rRNA* levels. All primer sequences are listed in Supplementary Table 1.

### Statistical analysis of qRT–PCR data

All qRT–PCR data were analyzed by Student’s *t*-test or 2-way ANOVA followed by post hoc Student’s *t*-test where appropriate. All statistical analysis and data plots were performed using Prism statistical software. Differences were considered significant when *P*-values were less than 0.05.

## Results and discussion

### Enteric infection upregulates the expression of metabolic genes

**Fig. 1.**
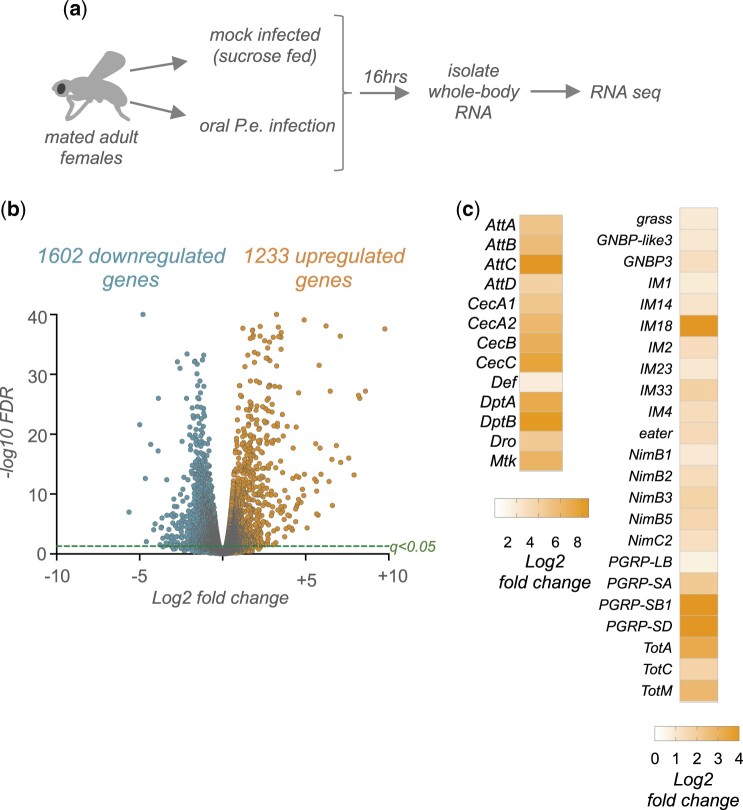
Enteric *P.e.* infection induces alterations in whole-body gene expression including upregulation of antimicrobial peptide genes. a) Schematic outline of our experimental approach. b) Volcano plot showing the gene expression changes following enteric infection. Genes were considered differentially expressed if they showed a significant [*q*-val (FDR corrected *P*-val) <0.05] change in expression that was >1.5-fold up- or downregulated in infected vs mock-infected flies. Dashed line indicates *q*-val = 0.05. c) Heatmap depicting the change in expression (Log2-fold change vs mock-infected flies) of antimicrobial peptide (AMP) genes and other immune-response genes following enteric infection.

We chose to examine whole-body transcriptome changes triggered by enteric infection. While this approach might limit our ability to detect subtle tissue-specific alterations in mRNA levels, we reasoned that it would be able to identify robust alterations in gene expression. Adult mated females were either mock-infected (fed with sucrose alone) or orally infected by feeding with *P.e.* We then isolated whole-body RNA at 16-h postinfection for RNA-seq analysis (Fig. 1a). Using a cut-off of ±1.5-fold and a false-discovery rate of *q* < 0.05, we identified 1,233 transcripts showing increased expression following infection and 1,602 transcripts showing reduced expression (Fig. 1b, Supplementary Table 1). The best-studied immune response to Gram-negative infection in *Drosophila* involves upregulation of the Imd/NF-Kappa B signaling pathway. Among the most strongly upregulated genes were the antimicrobial peptides (AMPs) which are known targets of the Imd pathway, and which function to reduce pathogen burden and promote resistance responses (Fig. 1c). We also saw induction of several other classes of genes encoding for secreted peptides and proteases that have also previously been shown to be induced upon bacterial infection and to mediate antimicrobial responses (Fig. 1c). These results confirm that our infection protocol induces a robust transcriptional immune response to enteric infection.

**Fig. 2.**
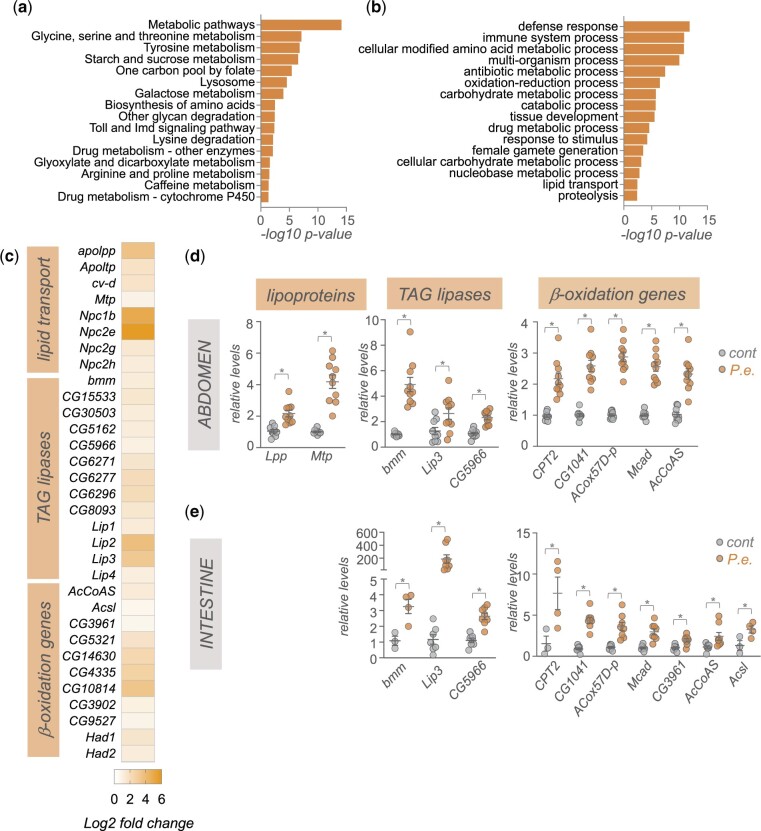
Enteric *P.e.* infection leads to upregulation of gut- and fat body-expressed lipid transport, TAG lipases, and beta-oxidation genes. a) KEGG pathway analysis of genes showing >1.5-fold increase following enteric infections. b) GO analysis (biological process categories) of genes showing >1.5-fold increase following enteric infections. c) Heatmap depicting the change in expression (Log2-fold change vs mock-infected flies) of lipid transport, TAG lipases, and beta-oxidation genes following enteric infection. Grey symbols show genes with strong enrichment in either the intestinal tissues or fat body, based on expression levels from FlyAtlas2. d, e) qPCR analysis of lipid transport, TAG lipases, and beta-oxidation genes from (d) isolated abdominal tissues or (e) intestines. Bars represent mean±SEM. Symbols represent individual data points, n = 6–10 per condition. *P < 0.05, Student’s *t*-test.

We then carried out KEGG and Gene Ontology analyses of the upregulated transcripts to help identify functional classes of genes whose expression is induced upon infection. As anticipated, KEGG pathway analyses showed enrichment in Toll and Imd signaling, while GO analysis showed the most significant enrichment was in the biological process categories of defense response and immune system process genes (Fig. 2, a and b). The other main classes of upregulated genes were, interestingly, related to metabolism. These included genes involved in lipid, carbohydrate, and amino acid metabolism (Fig. 2, b and c). Previous transcriptomic studies following enteric infection also showed changes in metabolic genes in specific tissues such as intestine, thoracic muscle, or abdominal adipose tissues (Buchon *et al.* 2009; Zhao and Karpac 2021). Similarly, transcriptome studies using models of systemic infection in flies also showed changes in metabolic gene expression (Clark *et al.* 2013; Troha *et al.* 2018). Our work adds to these studies to suggest that remodeling of host metabolism through altered gene expression is a common response to pathogenic bacterial infection in flies.

We examined the changes in metabolic gene expression by first exploring lipid metabolism genes. We saw increased expression of genes involved in lipid transport such as the Niemann-Pick type C family of cholesterol transporters, and the apolipoproteins, *Lpp*, *Ltp*, *cv-d*, and *Mtp* that are used to transport lipids from lipid storage tissues, such as the gut and abdominal adipose tissues, to other organs (Palm *et al.* 2012) (Fig. 2c). We also saw increased whole-body expression of TAG lipases and beta-oxidation genes (Fig. 2c). This is consistent with reports showing the depletion of lipid stores following enteric infection (Hang *et al.* 2014; Kamareddine, Robins, *et al.* 2018; Kamareddine, Wong, *et al.* 2018; Zhao and Karpac 2021; Deshpande *et al.* 2022). The main lipid storage tissues in the adult fly are the intestine, and the fat body and oenocytes, which are both enriched in the abdomen. We used qPCR to analyze gene expression in samples of isolated intestine and abdomen (abdominal tissues with intestine, ovaries, and Malpighian tubules removed) from mock-infected vs *P.e.*-infected flies. Consistent with our RNAseq analyses, we saw increased expression of the lipoproteins *Lpp* and *Mtp* in the abdominal samples (Fig. 2d). We also saw increased expression of both TAG lipases and beta-oxidation genes in both the intestine and abdominal samples (Fig. 2, d and e). Together these changes in gene expression suggest that upon enteric infection, flies alter their metabolism to mobilize fat and gut lipid stores and transport these lipids to other tissues to fuel metabolism through beta-oxidation. In fact, a recent report has described how differences in fat mobilization can explain interindividual differences in susceptibility to infection (Zhao and Karpac 2021). The gut and fat body are key tissues that coordinate host immune response to infection. They do so in large part by both increasing expression and release of the AMPs, and by initiating organ-to-organ signaling to coordinate whole-body host defences responses (Buchon *et al.* 2009). These effects rely on increased endocrine functions of both tissues which imposes a high protein synthetic and secretory burden (Martínez *et al.* 2020). As a result, both the gut and fat body likely have high metabolic demands following infections. Our RNA-seq and qRT–PCR data suggest that both tissues may upregulate fatty acid oxidation to sustain and support their metabolic needs. This is in line with emerging work in immunometabolism showing that many key immune system cells and tissues switch to fatty acid oxidation to meet their metabolic needs and to allow them to function properly to mediate defence responses (O’Neill *et al.* 2016; Cumnock *et al.* 2018; Batista-Gonzalez *et al.* 2019).

**Fig. 3.**
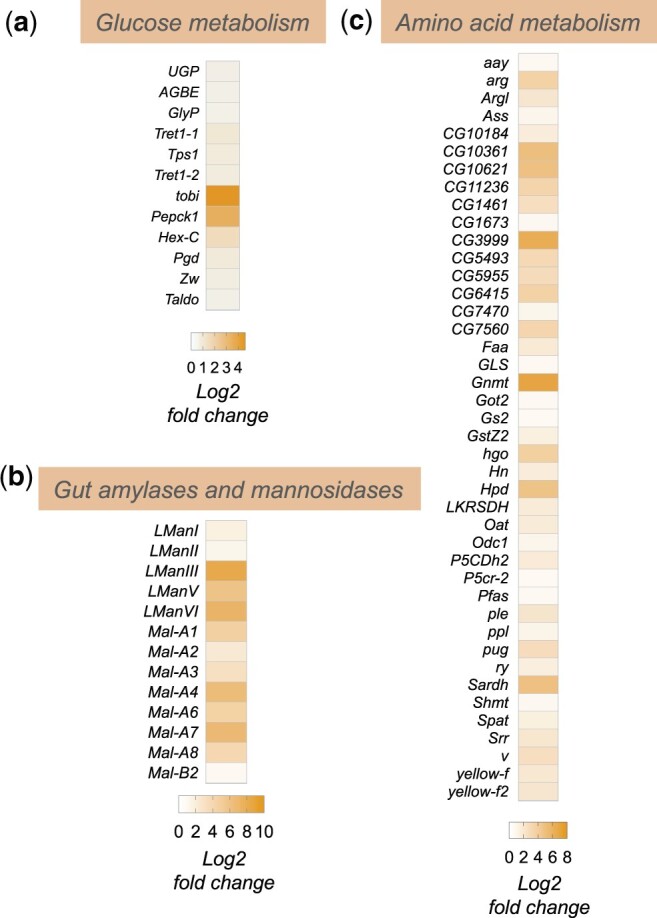
Enteric *P.e.* infection induces increase in sugar and amino acid metabolism genes. Heatmap depicting the change in expression (Log2-fold change vs mock-infected flies) of a) glucose metabolism, b) amylases and mannosidases, and c) amino acid metabolism genes following enteric infection.

In addition to alterations in lipid mobilization and lipid metabolic genes, we also saw that enteric infection led to an upregulation of whole-body expression of genes encoding regulators of both carbohydrate and amino acid metabolism (Fig. 3, a–c). For example, we saw increased expression of genes involved in both glycogen mobilization, and trehalose synthesis and transport (*GlyP, UGP, Tps1, Tret1-1, Tret1-2*) as well as increased expression of gluconeogenesis/glycolysis (*tobi, Pepck1, Hex-C*) and pentose phosphate pathway genes (*Pgd, Zw, Taldo*) (Fig. 3a). These changes suggest that flies remodel their glucose metabolism to adapt to infection. The upregulation of glycogen mobilization genes is consistent with previous reports showing depletion of glycogen stores following enteric infection (Hang *et al.* 2014; Deshpande *et al.* 2022). The muscle and fat body are the main glycogen storage tissues in flies, and one possibility is that the mobilized glucose may be used to fuel glycolysis and/or the pentose phosphate pathway in these tissues to help support their metabolic needs. In addition, the upregulation of trehalose synthesis genes suggests that some of the glucose may also be converted into trehalose, the main circulating form of glucose in flies, to be transported to other tissues.

Among the upregulated amino acid metabolism genes, two of the most strongly induced were *Gnmt* and *Sardh* (Fig. 3c). Both are involved in regulation of methionine metabolism and the control of S-adenosylmethione (SAM) levels. SAM is an important metabolite since it functions as a universal methyl donor in the control of methyltransferase activity and regulates antioxidant production. Interestingly, previous studies have shown that regulation of SAM levels in both the intestine and fat body play important roles in regulating physiology and tissue homeostasis. For example, alterations in SAM levels in the intestine have been shown to regulate levels of the Upd3 cytokine to control stem-cell mediated tissue renewal (Obata *et al.* 2018), which is known to be an important tissue repair response following enteric pathogen infection (Buchon *et al.* 2009; Jiang *et al.* 2009). In addition, alterations in SAM levels in the fat body have been shown to occur following necrotic wing injury and this alteration is important for controlling lipid levels and survival (Obata *et al.* 2014). Increased fat body expression of Gnmt and modulation of SAM levels can also extend lifespan (Obata and Miura 2015; Tain *et al.* 2020). Based on these previous reports, our findings suggest that modulation of intestine and/or fat body SAM levels may be an important metabolic response to enteric bacterial infection.

### The Imd pathway antagonizes the infection-mediated upregulation of metabolic gene expression

**Fig. 4.**
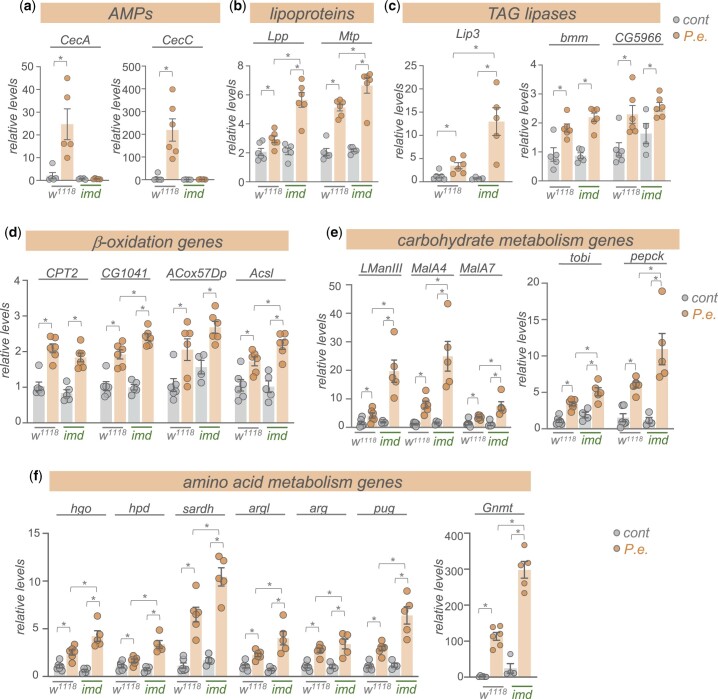
Enteric *P.e.* infection induces expression of metabolic genes independently of the Imd pathway. qRT–PCR analysis of mRNA levels of a) AMPs, b) lipoprotein genes, c) TAG lipases, d) beta-oxidation genes, e) carbohydrate metabolism genes, and g), amino acid metabolism genes from control (*w^1118^*) vs *imd* mutant adult females, that were either mock-infected (grey bars and symbols) or infected by oral feeding with *P.e.* for 24 h (orange bars and symbols). Bars represent mean±SEM. Symbols represent individual data points, n = 4–6 per condition. *P < 0.05, 2-way ANOVA, followed by Student’s *t*-test.

We next explored potential signaling and transcriptional mechanisms that might explain the upregulation in metabolic gene expression following enteric pathogen infection. We focused particularly on examining the conserved Imd/NF-Kappa B innate immunity pathway. The Imd pathway is the main signaling pathway induced by pathogenic Gram-negative infection in *Drosophila* (Kleino and Silverman 2014). The pathway is activated when cell-surface PGRP receptors detect bacterial peptidoglycans and stimulate a downstream intracellular signaling cascade involving Imd, a death-domain containing protein, that eventually leads to the activation and nuclear localization of the NF-Kappa B transcription factor, Relish (Kleino and Silverman 2014). One of the main transcriptional targets of Relish is the AMPs, which mediate the main antibacterial immune response to reduce pathogen load. We used qRT–PCR to compare whole-animal gene expression levels in control (*w^1118^*) or homozygous *imd* mutant animals that had either been mock-infected or orally infected by 24-h feeding with *P.e.* We saw that the infection-mediated upregulation of 2 AMPS, *CecA* and *CecC* seen in control (*w^1118^*) animals was completely suppressed in the *imd* mutant animals, confirming that this line is a strong loss-of-function of the Imd pathway (Fig. 4a). We then analyzed expression of representative metabolic genes from several main classes: lipoproteins, TAG lipases, beta oxidation genes, carbohydrate metabolic genes, and amino acid metabolic genes. From these analyses, 3 main findings emerged (Fig. 4, b–f). First, we found that infection induced a strong upregulation of all the metabolic genes that we tested, thus confirming the transcript changes that we saw in our RNA-seq analyses. Second, we saw that none of these increases in metabolic gene expression were blocked in the *imd* mutants. Thus, in contrast to the induction of the AMPs, the increase in metabolic gene expression does not rely on the Imd/NF-Kappa B immune signaling pathway. Thirdly, and most interestingly, we saw that in many cases, the increase in metabolic gene expression seen following infection was further exacerbated in the *imd* mutant flies. These results suggest that the Imd pathway may function to antagonize the infection-mediated changes in host metabolism. Indeed, a previous report showed that constitutive genetic activation of the Imd pathway in larval fat body led to downregulation of many carbohydrate and lipid metabolic genes, including several that we see are induced upon infection and further increased in infected *imd* mutants (Davoodi *et al.* 2019). In addition, Relish, the transcriptional effector of the Imd pathway, has been shown to antagonize the starvation-mediated induction of the lipase, *bmm* (Molaei *et al.* 2019). Moreover, Relish knockdown has been shown to exacerbate the infection-induced increase in amino acid metabolism genes in muscle, including Gnmt, and the extent of these muscle effects of Relish were shown to underlie interindividual differences in infection susceptibility (Zhao and Karpac 2021). These previous reports together with our RNA-seq and qPCR results suggest that while induction of the Imd pathway is necessary to induce AMPs and induce antibacterial responses, it also functions to limit induction of metabolic genes.

What signaling pathways and transcriptional mechanisms, if not the Imd pathway, may lead to the upregulation of metabolic gene expression following infection? One possibility is the endocrine insulin/PI3K/TOR pathway. We previously showed that this pathway is induced upon enteric infection with *P.e.* independently of Imd signaling and we found that this induction was needed to promote lipid synthesis gene expression (Deshpande *et al.* 2022). Similarly, Charroux and Royet (2022) showed that infection induces an upregulation of SREBP in the fly fat body through insulin/PI3 kinase signaling. Thus, given that the insulin/P3K/TOR pathway is the one of the main regulators of organismal metabolism in flies, many of the increases in lipid, carbohydrate, and amino acid metabolism genes that we see maybe mediated through this pathway. Furthermore, since Imd signaling has been shown to negatively regulate insulin/PI3K signaling (Davoodi *et al.* 2019), this may also explain, in part, why the increased expression of metabolic genes is further exacerbated in *imd* mutants. It may seem paradoxical that insulin/PI3K/TOR signaling would induce both lipid synthesis and lipolysis genes. However, it is possible that these effects may be occurring in a cell- or tissue-specific manner. For example, activation of the PI3K/TOR pathway in oenocytes has been shown to reduce lipid levels in these cells, but then lead to an increase in lipid levels in fat body cells (Ghosh *et al.* 2020). PI3K and TOR signaling are known to exert tissue-specific effects on metabolism. Hence, future studies using tissue-specific genetic inhibition of PI3K/TOR signaling will be informative to establish if these signaling pathways mediate some of the infection-mediated changes in metabolic gene expression that we identified in our RNA-seq analyses, and to determine whether any of these changes are tissue-specific.

### Enteric infection downregulates the expression of many CNS and intestinal signaling pathways and transcription factors

**Fig. 5.**
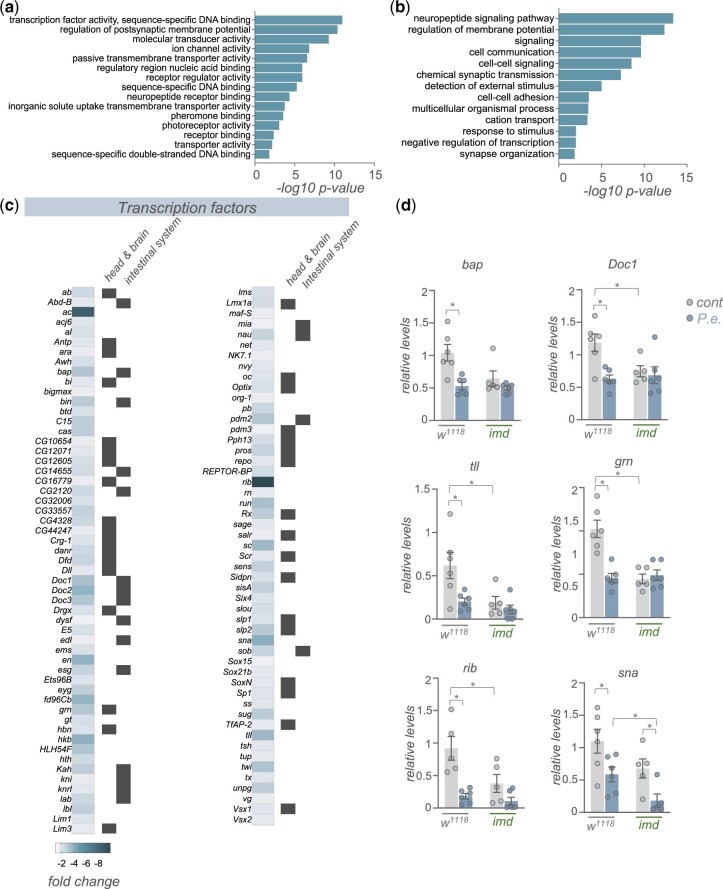
Enteric *P.e.* infection downregulates the expression of many transcription factor genes. a, b) GO analysis (a, biological process categories, b, molecular function categories) of genes showing >1.5-fold increase following enteric infections. c) Heatmap depicting the change in expression (Log2-fold change vs mock-infected flies) of transcription factor genes following enteric infection. Grey symbols show genes with strong enrichment in either the head/brain or intestinal system, based on expression levels from FlyAtlas2. d) qPCR analysis of selected transcription factor mRNA levels from control (*w^1118^*) vs *imd* mutant adult females, that were either mock-infected (grey bars and symbols) or infected by oral feeding with *P.e.* for 24 h (blue bars and symbols). Bars represent mean±SEM. Symbols represent individual data points, n = 4–6 per condition. * *P* < 0.05, 2-way ANOVA, followed by Student’s *t*-test.

We saw that 1,602 transcripts showed reduced mRNA expression following infection (Fig. 1b). GO term and KEGG pathway analyses of these genes, interestingly, revealed enrichment in 2 broad classes of genes—transcription factors and cell–cell signaling pathways (Fig. 5, a and b). We found that ∼100 transcription factors showed reduced expression following infection (Fig. 5c). We used FlyAtlas2 (Leader *et al.* 2018) to explore where each of these transcription factors is normally expressed, and we saw that many of them show enriched expression either in the intestinal system (crop, midgut and/or hindgut) or the head and brain (head, eye, brain/CNS, and/or thoracicoabdominal ganglion) (Fig. 5c). We also selected a few genes that showed strong changes in expression in the RNA-seq analysis and examined whether their downregulated expression was mediated through the Imd pathway by using qRT–PCR to compare infection-mediated changes in whole-body expression in control (*w^1118^*) vs *imd* mutants. We saw that for each of the 6 transcription factors that we examined (*bap, Doc1, tll, grn, rib*, and *sna*) mRNA expression levels were significantly reduced following infection, thus confirming the transcript changes that we saw in our RNA-seq analyses (Fig. 5d). We also saw that basal expression levels of each transcription factor were significantly lower in mock-infected *imd* mutants compared to controls, and that infection did not decrease these levels further (except for *sna*) (Fig. 5d). These results suggest that the infection-mediated decreases in expression of these transcription factors is not mediated through induction of Imd signaling, and instead Imd signaling is needed to maintain basal expression of these genes.

**Fig. 6.**
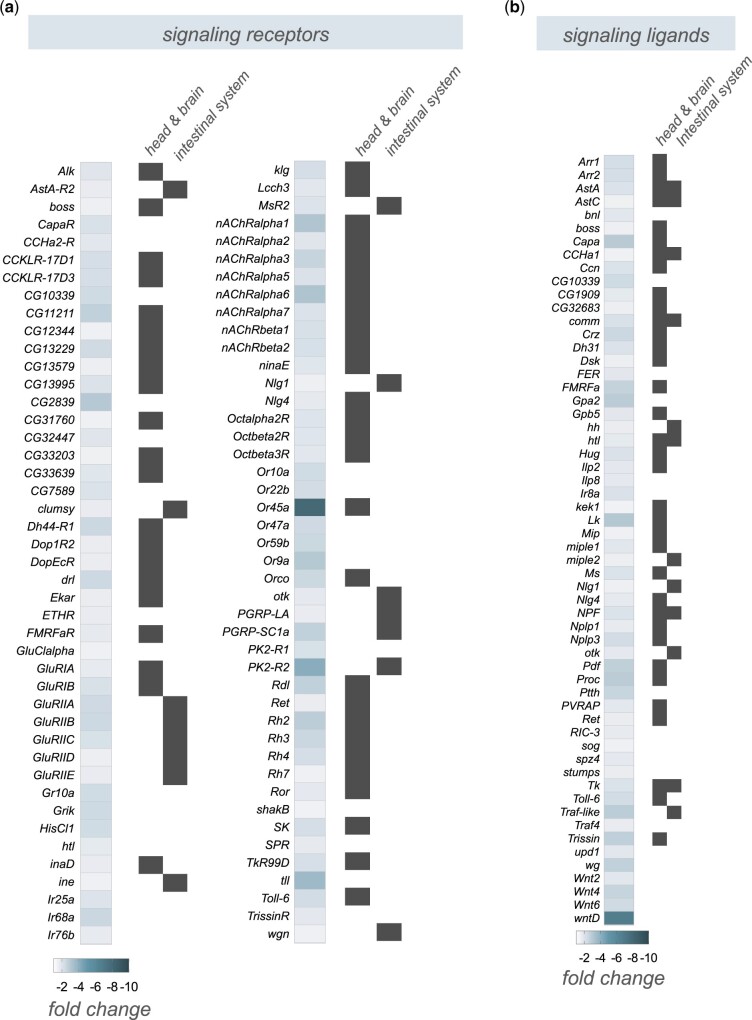
Enteric *P.e.* infection downregulates the expression of many signaling receptors and ligands that show brain- and intestine-enriched expression. a, b) Heatmap depicting the change in expression (Log2-fold change vs mock-infected flies) of (a) signaling receptors and (b) signaling ligands following enteric infection. Grey symbols show genes with strong enrichment in either the head/brain or intestinal system, based on expression levels from FlyAtlas2.

**Fig. 7.**
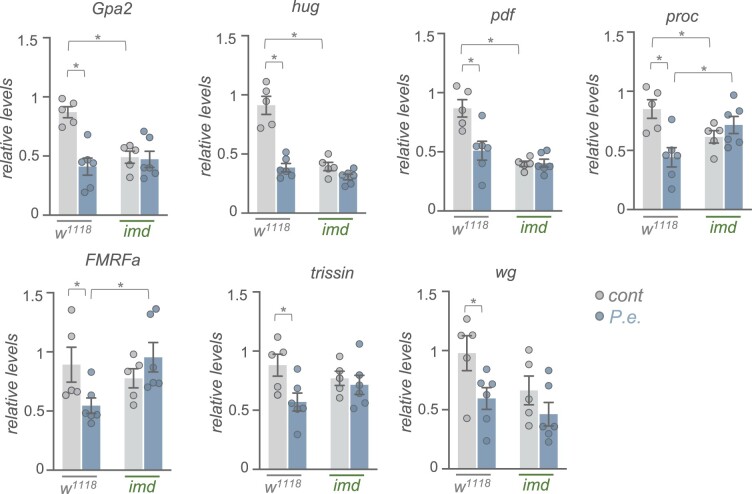
Enteric *P.e.* infection downregulates the expression of signaling molecules independently of Imd signaling. qPCR analysis of selected signaling molecule mRNA levels from control (*w^1118^*) vs *imd* mutant adult females, that were either mock-infected (grey bars and symbols) or infected by oral feeding with *P.e.* for 24 h (blue bars and symbols). Bars represent mean±SEM. Symbols represent individual data points, n = 4–6 per condition. *P < 0.05, 2-way ANOVA, followed by Student’s *t*-test.

Another large group of genes that showed reduced expression following infection are those involved in cell–cell signaling (Fig. 5, a and b). These include both cell surface receptors (Fig. 6a) and secreted peptide ligands (Fig. 6b). In some cases (e.g. *AstA, Capa, FMRFa, Ms, Trissin, Tk*), we found expression of both the secreted factors and as well as their corresponding receptors were coordinately decreased, suggesting downregulation of signaling through pathways coupled to these ligand/receptor pairs. Also, as with the downregulated transcription factors, we found that most downregulated receptors and ligands showed enriched expression in the head/CNS and/or intestinal system as indicated by FlyAtlas2 expression profiles (Fig. 6, a and b). We selected several of these signaling peptides (*Gpa2, hug, pdf, proc, FMRFa, trissin, wg*) that showed strong changes in expression in the RNA-seq analysis to examine whether the infection-mediated decrease in expression was dependent on the Imd signaling pathway. Using qRT–PCR analysis, we found that the whole-body expression of each of these genes was decreased following enteric infection with *P.e.*, consistent with our RNA-seq analyses (Fig. 7). For three of these genes (*Gpa2, hug*, *and pdf*) the expression levels in both the mock-infected and infected *imd* mutants were significantly lower than the control mock-infected flies, suggesting that the infection-mediated downregulation of these genes did not occur through increased Imd signaling (Fig. 7). However, for 2 genes (*proc* and *FMRFa*), both of which show strongly enriched neuronal expression, the infection-mediated decreases in mRNA expression were reversed in the *imd* mutants, suggesting that the suppression of these genes may be mediated through Imd signaling (Fig. 7). Indeed, Imd signaling has been shown to be induced in neurons through direct effects of peptidoglycan on the brain (Kurz *et al.* 2017).

Our RNA-seq results showed decreased expression of a broad range of transcription factors and cell–cell signaling molecules. Given that the normal tissue expression patterns of these genes from FlyAtlas show that most are enriched in the brain and/or intestine, we can speculate from our RNA-seq results that gut infection leads to: (1) a direct downregulation of transcription factor and signaling molecule expression within the intestine, and/or (2) an indirect, nonautonomous decrease in transcription factor and signaling molecule expression within the brain. The former scenario could reflect infection-induced alterations in gut-to-brain signaling. For example, several of the gut-enriched signaling peptides, such as *AstC, CCHa1, NPF*, and *Tk* are expressed in enteroendocrine (EE) cells, a population of secretory cells in the gut, and they have receptors in the brain, suggesting they may signal from the gut-to-brain to mediate changes in gene expression. Indeed, a direct gut-to-brain signaling role has been shown for *NPF, AstC*, and *Dh31* in the context of nutrient regulation of feeding and metabolism (Yoshinari *et al.* 2021; Kubrak *et al.* 2022; Lin *et al.* 2022). Interestingly, a recent paper showed that enteric infection with pathogenic bacteria, such as *P.e.*, led to an increase in EE cells (Liu *et al.* 2022), suggesting that the reduced levels of EE-expressed signaling peptides that we saw did not arise due to damage-induced loss of these cells. In the latter scenario, it is possible that the infection-mediated reductions in signaling molecules and transcription factors occur mostly or exclusively in the brain. In this case, infected intestines may signal to the brain to alter neuronal gene expression. For example, upon enteric bacterial infection, damaged gut epithelial cells have been shown to secrete the cytokine, Upd2, which signals to the brain to modulate glial gene expression (Cai *et al.* 2021).

Several of the changes in both transcription factor levels and intestinal and/or brain signaling could also explain the increases in metabolic gene expression that we observed. For example, both *sug* and *REPTOR-BP* (both of which we see are downregulated upon infection) have been shown to regulate the expression of both carbohydrate and lipid metabolic genes (Mattila *et al.* 2015; Tiebe *et al.* 2015). Interestingly, *REPTOR-BP*-dependent transcription is negatively regulated by insulin/TOR signaling (Tiebe *et al.* 2015), which would be consistent with some of the changes in metabolic gene expression being mediated though upregulated insulin/PI3K/TOR signaling. Furthermore, several of the intestinal and neuronally expressed signaling molecules, such as *NPF* (Yoshinari *et al.* 2021), *Tk* (Song *et al.* 2014; Kamareddine, Robins, *et al.* 2018), *AstC* (Kubrak *et al.* 2022), and *Crz* (Kubrak *et al.* 2016), have been shown to alter whole-body metabolism (Zhou *et al.* 2020; Medina *et al.* 2022).

One other possibility is that the changes in both intestinal and neuronal signaling molecules that we observed may mediate alterations in fly behavior upon infection. It is known that infected flies will alter their feeding behavior, often to avoid eating infected food (Soldano *et al.* 2016; Charroux *et al.* 2020; Cai *et al.* 2021), and they will alter their fecundity and egg-laying behavior (Masuzzo *et al.* 2019, 2020), perhaps to limit the energetically costly process of reproduction while fighting infection (Schwenke *et al.* 2016). Given that several of the neuropeptide signaling molecules and pathways that we see downregulated upon infection are known to affect both feeding and reproduction (Schoofs *et al.* 2017; Ameku *et al.* 2018; Nassel and Zandawala 2019; Hadjieconomou *et al.* 2020; Kim *et al.* 2021; Sadanandappa *et al.* 2021), it is possible that they may modulate either process to help promote infection tolerance. Thus, enteric infection-mediated changes in both gut-to-brain signaling and CNS signaling may be a general mechanism to coordinate multiple whole-body physiological and behavioral responses to infection.

One limitation of our study is that we used whole-body analysis of gene expression in our RNA-seq analyses. Future experiments using tissue-restricted mRNA measurements and genetic manipulations of gene function will be informative in defining whether any of the differentially expressed genes that we identified may be mediating tissue-specific effects on metabolism and physiology in infected flies. Another limitation of our work is that we examined gene expression changes upon infection only in females, and not in males. Interestingly, a recent study using a model of systemic bacterial infection in flies described sex differences in Imd signaling and AMP expression, but not metabolism upon infection (Vincent and Dionne 2021). Thus, it will be interesting to examine whether there are any sex differences in the enteric infection-mediated changes in gene expression and the modulation of these changes by Imd signaling that we see in females.

## Supplementary Material

jkac163_Supplemental_Table_1

jkac163_Supplemental_Table

## Data Availability

The RNA-sequence data have been deposited in NCBI’s Gene Expression Omnibus and are accessible through GEO Series accession number GSE202578 (https://www.ncbi.nlm.nih.gov/geo/query/acc.cgi?acc=GSE202578). Supplemental material is available at G3 online.
