# Supplementary material for: Enteric bacterial infection in Drosophila induces whole-body alterations in metabolic gene expression independently of the immune deficiency signaling pathway
Source: G3 (Bethesda). 2022 Jul 4;12(11):jkac163. doi: 10.1093/g3journal/jkac163 (PMC9635644; doi:10.1093/g3journal/jkac163)
Supplement: jkac163_Supplemental_Table [file jkac163_supplemental_table.zip › jkac163_Supplemental_Table_Legends.docx]

**Supplemental Table 1.** Processed RNA-seq data.

**Supplemental Table 2.** List of primers used in this study.
