# Supplementary material for: Enteric bacterial infection in Drosophila induces whole-body alterations in metabolic gene expression independently of the immune deficiency signaling pathway
Source: G3 (Bethesda). 2022 Jul 4;12(11):jkac163. doi: 10.1093/g3journal/jkac163 (PMC9635644; doi:10.1093/g3journal/jkac163)
Supplement: jkac163_Supplemental_Table [file jkac163_supplemental_table.zip › jkac163_Supplemental_Table_2.pdf]

Gene	Forward primer	Reverse primer
Acetyl Coenzyme A synthetase (AcCoAS)	CGTGGAGAGAAACCCCTGTA	TTTCCTCCTCGACATGATCC
Medium-chain acyl-CoA dehydrogenase (mcad)	ACGGCTTCAACAGCGAGTAT	ACCCTTAGCAGCCTCGTACA
Acyl-Coenzyme A oxidase at 57D proximal (ACoX57Dp)	AAGGCCGGTGATTACTTGTG	GGAGTCCATGGAGCAGGTAA
Carnitine palmitoyltransferase 2 (CPT2)	AACTTCGAAATCGACGATGC	TCCGGACTTAGGCCATACTG
Carnitine O-acetlytransferase (CG1041)	GGCAGCAAGGAGAAGAATTG	AGCACGTGAGTCCCTGAAGT
Acyl-CoA synthetase long-chain (Acsl)	CAAAGTCAAGTGGGTGCAGA	CTGAATCAGCTCCAAGCACA
Apolpp (lpp)	ATCGGCTCAACACAAAAACC	AGGCAAAAGCGATCTCAAAA
ApoMtp (Mtp)	GTGGGAAGCTTCGTGAAGAG	AAAACGCGATACCATTCGAG
Brummer	CAATAAGGGTCTGGCCAACTGGAT	TAAGTCCTCCACCATTACTCTGGC
Lipase 3	GGAACTTCAGCTGGAACGAG	GGCGGACTTGATCTTGTCAT
CG 5966	CTCGCAGTGTCCTTTCCTTG	TGCTCCTGGTAATCCTCCTG
Cecropin A	TCTTCGTTTTCGTCGCTCTCA	ATTCCCAGTCCCTGGATTGTG
Cecropin C	TCATCCTGGCCATCAGCATT	CGCAATTCCCAGTCCTTGAAT
LManIII	GACTTGGGTTGGCTAAAAACGG	CGACGTGAGTCCTTGCTCAG
Mal-A4	TTCAAAGACAGTGACGGAAATGG	TTTCAGAAAAGGAGACAGCCAG
Mal-A7	TTCAGCCGGAATACGGAACAC	GTGATTGGGTACGAAATCCAGAA
pepck1	TGATCCCGAACGCACCATC	CTCAGGGCGAAGCACTTCTT
tobi	GTGCGACTTAGGAGAGGTGAT	GCTGTCAAAGTTGTCGAAGGT
5S rRNA	ACGACCATACCACGCTGAAT	AGCGGTCCCCCATCTAAGTA
Gnmt	GATGCAAGCGTGTCCTAGAC	CTCAATGACCCACTTATCGAAGG
hgo	AAGTACCTCTCGGGTTTTGGG	GCATAGAGTTTGTATGGGCACAC
Hpd	ATTCGTGAGATTCGCTACCATC	GGCAATCCGTTTAGAAGGACATC
Sardh	CGCCGTGGAATCAGCAAAC	TGATACAGTGTGTGACAGCCC
Faa	AGTGCCAGAGAACAGCGATTT	GTCCAGGACATGCTCTCCAA
argl	GAGCTGTTGCGATTCGATTGG	CGCCGGTAATCTCCACCAG
arg	GGTGAAACTCATGCTCGTGGA	ACACCAGGGACAAATTCGGC
pug	GACCTTGGTGGTTTCCTGCC	GTTGCGGGTCTTTGAGTGG
bap	GGCAACCCAGCAACTATCTG	CAATTTGCGCTGCATGTAGTC
Doc1	GAGACGGAGTTCGTAGCTGTC	GACTCGCGGAAACCCTTAGC
grn	TGGCCTGTACTACAAAATGAACG	TGTGGTTGTGGTCTTGCAGTT
rib	GTGGACGATGAACAGTTCCAG	GCAATCTCGAAGCCCTTCACT
sna	ATGGCCGCCAACTACAAAAG	GCAAACTGTGAGTCCTTGGTC
tll	TCGGAGGGTTCACCAGACAT	GCACATGGTATAGAATGCGACT
FMRFa	CATGCACTTCGGCAAGAGG	GCCATACCGATCCATAGCTGC
Gpa2	CCAATCTCAACAGTTGCCGAG	GCCAAGCGTCCTTTCCCAT
Hug	CGCCACTGACCTATTACCTGC	CGTCCAGTAGTCGCCAGGA
Pdf	GCTCGCTACACGTACCTTGTC	ACGTTGTTGAACCAGTCGAGG
Trissin	GCTCACTTCCAGATCATCTTGC	TGCGAAGGTAGTTAAAGCAGC
wg	CCAAGTCGAGGGCAAACAGAA	TGGATCGCTGGGTCCATGTA
Proc	TTGGTTTGGATGACGGTGCT	CTGTTGTGGGTCCTCATTGC

Supplemental Table 1. List of primers used in this study
